# Study on the Binary Hydraulic Kinetics Model of Glass Powder-Cement: Numerical Simulation

**DOI:** 10.3390/ma16051957

**Published:** 2023-02-27

**Authors:** Yang Ming, Ling Li, Hao Ren, Ping Chen, Xuandong Chen

**Affiliations:** 1 Guangxi Key Laboratory of New Energy and Building Energy Saving, Guilin 541004, China; 2 College of Civil and Architecture Engineering, Guilin University of Technology, Guilin 541004, China; 3 Guangxi Engineering and Technology Center for Utilization of Industrial Waste Residue in Building Materials, Guilin 541004, China; 4 Collaborative Innovation Center for Exploration of Nonferrous Metal Deposits and Efficient Utilization of Resources, Guilin 541004, China

**Keywords:** cement hydration, numerical simulation, glass powder, hydration kinetics model, hydration heat evolution, optimum glass powder

## Abstract

As supplementary cementitious material, glass powder has been widely used in concrete, and many investigations on the mechanical properties of glass powder concrete have been carried out. However, there is a lack of investigations on the binary hydration kinetics model of glass powder-cement. Based on the pozzolanic reaction mechanism of glass powder, the purpose of this paper is to establish a theoretical model of the binary hydraulic kinetics model of glass powder-cement to investigate the effect of glass powder on cement hydration. The hydration process of glass powder-cement mixed cementitious materials with different glass powder contents (e.g., 0, 20%, 50%) was simulated using the finite element method (FEM). The numerical simulation results are in good agreement with the experimental data of hydration heat in the literature, which verifies the reliability of the proposed model. The results show that the glass powder can dilute and accelerate the hydration of cement. Compared to the sample with 5% glass powder content, the hydration degree of the glass powder decreased by 42.3% for the sample with 50% glass powder content. More importantly, the reactivity of the glass powder decreases exponentially with the increase in the glass particle size. In addition, the reactivity of the glass powder tends to be stable when the glass particle size is greater than 90 μm. With the increase in the replacement rate of the glass powder, the reactivity of the glass powder decreases. When the replacement rate of the glass powder is greater than 45%, the concentration of CH reaches a peak at the early stage of the reaction. The research in this paper reveals the hydration mechanism of glass powder and provides a theoretical basis for the application of glass powder in concrete.

## 1. Introduction

The cement manufacturing industry is a major cause of global warming [1,2,3]. Moreover, with the increasingly mature preparation technology of glass products, glass products are widely used in construction, life and other aspects, and waste glass accounts for about 7% of global solid waste every year [1]. In recent years, a large number of experimental studies have been carried out on the application of waste glass in concrete [2,3,4,5,6,7,8]. Adesina et al.’s [9,10] experiments in the incorporation of glass powder as the precursor in alkali-activated materials resulted in an improvement in the workability and extension of the set times. Moreover, the pozzolanic reaction mechanism of glass powder and the evolution of the micro-pore structure were also investigated. However, the theoretical investigation on the binary hydration kinetic model of glass powder-cement is rarely reported, which has led to the failure to systematically and quantitatively study the influence of glass powder content and particle size on the hydration of cement and glass power. Based on the reaction-diffusion theory, Ravi A. Patel et al. [9] used the lattice Boltzmann method to study the change in the pore microstructure of ordinary Portland cement slurry during the calcium leaching process. Based on the VCCTL platform, more than 20 hydration reactions of different types of cement have been carried out by B.E. Watts et al. [10], revealing the change rules of the hydration heat and compressive strength of cement. Bentz et al. [11] built the hydration model of the cement-limestone binary blend system to study the dilution action, nucleation and chemical effect of limestone addition. Tomosawa et al. [12,13,14] established the cement hydration shrinkage core model based on the cement hydration delay effect, reaction effect and diffusion effect. Wang Xiaoyong et al. [15,16,17] established multiple composite hydration models of slag-cement, fly ash-cement, limestone-cement-fly ash, etc., on the basis of the shrinking core model. With the maturity of cement hydration theory, a series of mature cement hydration software have been formed, such as HYMOSTRUC, CEMHYD3D, μic, Du-COM, VCCTL, HydratiCA, etc. The software can simulate the process of cement hydration and reveal the microstructure evolution of the cement hydration, which is of great significance to the study of the cement hydration mechanism. However, the theoretical model and software are only for Portland cement, fly ash cement and slag cement hydration, without considering the effect of glass powder on cement hydration. Therefore, it is very necessary to build a Binary Hydraulic Kinetics Model of Glass Powder-Cement to simulate the hydration process of glass powder concrete.

Similar to slag and fly ash, a large amount of the active silica in glass powder reacts with calcium hydroxide in pore solution to form a pozzolanic reaction. In this paper, based on the pozzolanic reaction mechanism of glass power, a theoretical model of the binary hydraulic kinetics model of glass powder-cement is established by using the theory of shrinkage core hydration, which is analogous to the hydration reaction of Wangxiaoyong et al. [15,16,17] in slag and fly ash-cement. The hydration process of glass powder-cement mixed cementitious materials with different glass powder content was simulated using the finite element method (FEM). The experimental data are in good agreement with the numerical simulation data, which shows that the numerical model is reliable. Furthermore, the dilution effect of glass powder on the hydration of water and the influence of different particle sizes and the content of glass powder on the reactivity of glass powder were studied. It is worth noting that the use of glass powders in the technology of cement composites fits well into sustainable development, which is highly expected in every area of our lives. The research in this paper provides theoretical support for the application of glass powder in cement-based materials and has positive significance for guiding the experiment.

## 2. Binary Hydraulic Kinetics Model of Glass Powder-Cement

### 2.1. Hydration Mechanism

**Figure 1:**
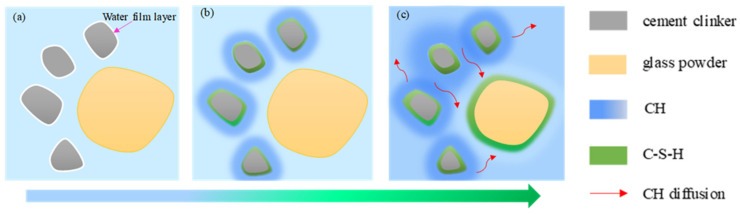
Evolution of glass powder-cement hydration process (**a**) initial mixing (**b**) cement hydration (**c**) pozzolanic reaction of glass powder.

The hydration of a glass and cement mixture is more complicated than that of single cement. The pozzolanic effect and microaggregate effect of glass powder affects the hydration process and microstructure. The schematic diagram of glass the powder-cement binary hydration process is shown in Figure 1. In the initial stage of mixing water, cement and glass power, a thin water curtain is formed on the surfaces of the glass power and the cement to hinder the diffusion of the water into the cement and to delay the hydration of the cement and the pozzolanic reaction of the glass powder. As the water curtain on the cement surface is absorbed by the cement, the external water diffuses into the cement and chemically reacts with the cement clinker. Papadakis et al. [18,19] proposed the chemical formula of cement hydration by analyzing the chemical composition of cement clinker, as shown in Formulas (1)–(4). During hydration, C-S-H deposits are gradually formed around the cement, and the generated CH diffuses into the solution. The hydration rate is controlled by the diffusion rate and reaction rate. The pozzolanic reaction of glass powder is that the active silicon dioxide in the glass powder reacts with CH in the solution to generate C-S-H. At the initial stage of the hydration reaction, the cement just begins to hydrate, and the solubility of the CH in the solution is relatively low, so the pozzolanic reaction of the glass powder does not occur at this stage, as shown in Figure 1b. With the continuous hydration of the cement, the concentration of CH in the solution increases continuously, and the glass powder begins to react with the CH in the solution, and forms C-S-H deposits around the glass powder. At the same time, the high concentration CH around the cement particles also moves to the low concentration CH area around the glass powder particles, so that the pozzolanic reaction can be continued, and the pozzolanic reaction is shown as Formula (5).

(1) $$2C_{3}S+6H\to C_{3}S_{2}H_{3}+3CH$$

(2) $$2C_{2}S+4H\to C_{3}S_{2}H_{3}+CH$$

(3) $$C_{3}A+C\bar{S}H+10H\to C_{4}A\bar{S}H_{12}$$

(4) $$C_{4}AF+2CH+10H\to C_{6}AFH_{12}$$

(5) $$mCa{\left(OH\right)}_{2}+Si_{2}O+nH_{2}O\to mCaO\cdot Si_{2}O\cdot nH_{2}O$$

### 2.2. Cement Hydration Model

There are many theoretical models of cement hydration, each of which has its own advantages and disadvantages. In this paper, the shrinking core model, proposed by Tomosawa et al. [12], is adopted as the cement hydration model. Park et al. [13], Maruyama et al. [14] and Wang et al. [15,16,17,20,21,22] have revised and developed the shrinking core model. In the model, the delay effect of the water curtain on the reaction in the initial process, the hydration reaction rate, and the influence of the water diffusion factors in C-S-H cement on cement hydration can be expressed by the following equation:

(6) $$\frac{d\alpha }{dt}=\frac{3\left(S_{w}/S_{0}\right)\rho_{w}C_{w-free}}{\left(v+w_{g}\right)r_{0}\rho_{c}}\frac{1}{\left(\frac{1}{k_{d}}-\frac{r_{0}}{D_{e}}\right)+\frac{r_{0}}{D_{e}}{\left(1-\alpha \right)}^{\frac{-1}{3}}+\frac{1}{k_{r}}{\left(1-\alpha \right)}^{\frac{-2}{3}}}$$

where α is the degree of hydration, $r_{0}$ is the initial radius of cement,v is the stoichiometric ratio of water to cement measured by a mass meter, taking 0.25, $w_{g}$ is the physically combined water in the hydration products, taking 0.15, $\rho_{c}$ is the mass density of cement, $\rho_{w}$ is the mass density of water, $C_{w-free}$ is the amount of capillary water outside the hydration product, the calculation formula is

(7) $$C_{w-free}={\left(\frac{W_{0}-0.4\times \alpha \times C_{0}}{W_{0}}\right)}^{\xi }$$

$k_{d}$ is the cement hydration reaction coefficient [20], the calculation formula is

(8) $$k_{d}=\frac{B}{\alpha^{1.5}}+C\alpha^{3}$$

$D_{e}$ is the diffusion coefficient of water in C-S-H [14], the calculation formula is

(9) $$D_{e}=D_{e0}\ln\left(\frac{1}{\alpha }\right)$$

### 2.3. Pozzolanic Reaction of Glass Powder

The main component of the glass is amorphous silicon dioxide, and when the particle size of the glass powder is small enough, the amorphous silicon dioxide shows pozzolanic activity and performs the pozzolanic reaction with the alkaline pore solution. The pozzolanic reaction model of glass powder is established by the shrinking core model, which is similar to the shrinking core model of the cement hydration reaction. The pozzolanic reaction of glass powder, slag and fly ash with water mud mainly refers to the reaction of active silicon dioxide in mineral composition with CH to form C-S-H. However, the content of silicon dioxide in the three materials is different, and the other mineral compositions are also different. The main component of glass powder is silicon dioxide. The pozzolanic reaction of the glass powder also involves three processes: the initial delay process, surface reaction process and water diffusion process. In the initial stage, the CH concentration is low and there is a water curtain on the surface of the glass powder, so the delay process is dominant. Subsequently, the surface reaction process and the water diffusion process are dominant. At the same time, there is an obvious difference between the pozzolanic reaction of glass powder and the cement reaction [23,24]; cement hydration produces CH, while the pozzolanic reaction of glass powder consumes CH, so the CH concentration has a significant influence on the pozzolanic reaction. The pozzolanic reaction model of glass powder is established by analogy with the pozzolanic reaction model of fly ash and slag proposed by Wang et al. [17,20].

(10) $$\frac{d\alpha_{glass}}{dt}=\frac{m_{CH}\left(t\right)}{P_{g}}\cdot \frac{W_{cw}}{W_{0}}\cdot \frac{3}{v_{glass}r_{0glass}\rho_{glass}}\cdot \frac{1}{\left(\frac{1}{k_{dglass}}-\frac{r_{0glass}}{D_{eglass}}\right)+\frac{r_{0glass}}{D_{eglass}}{\left(1-\alpha_{glass}\right)}^{\frac{-1}{3}}+\frac{1}{k_{rglass}}{\left(1-\alpha_{glass}\right)}^{\frac{-2}{3}}}$$

where $P_{g}$ is the content of glass powder, $m_{CH}\left(t\right)$ is the CH content in the well solution, $v_{glass}$ is the stoichiometric coefficient of the glass frit chemical reaction; $a_{glass}$ is the stoichiometric coefficient of the glass frit chemical reaction; $k_{dglass}$, $D_{eglass}$, $k_{rglass}$ are the delay effect factor, the diffusion effect factor and the reaction effect factor, respectively, and their expressions are similar to those of cement hydration. $W_{cw}$ is the capillary water mass.

### 2.4. Hydration Couple of Glass Powder and Cement

Under the condition that the water-binder ratio of water is certain, when the glass powder replaces a part of the cement, the glass powder plays a role in diluting the cement, so that the mass ratio of water to cement is increased, and the hydration of the cement is accelerated. At the same time, the CH generated by cement hydration is partly consumed by the pozzolanic reaction of the glass powder. At the same time, the pozzolanic reaction will consume part of the capillary water and generate some chemically combined water. Maekawa et al. [25] measured the contents of the consumed CH, capillary water and chemical water generated by the pozzolanic reaction of slag and fly ash through experiments. Due to the different contents of silicon dioxide in glass powder, slag and fly ash, the contents of CH consumed by the pozzolanic reaction of glass powder, and the capillary water and chemical water generated, are calculated according to the content ratio of silicon dioxide in this paper. The relevant specific values are given in Table 1, and the average value is taken in this paper. From the stoichiometric coefficients and the conservation of mass [15,16,17], the CH, $W_{cw}$, $W_{cbw}$ can be calculated, and the total hydration heat Q can be calculated according to the hydration heat of the unit cement and glass powder [12]. The relevant calculation is shown in Formulas (11)~(14).

(11) $$m_{CH}\left(t\right)=\left(0.49\cdot g_{c3s}+0.22\cdot g_{c2s}-0.3\cdot g_{c4af}\right)\cdot C_{0}\cdot a-0.24\cdot P_{g}\cdot a_{glass}$$

(12) $$w_{cw}\left(t\right)=w_{0}-0.42\cdot C_{0}\cdot a-0.18\cdot P_{g}\cdot a_{glass}-0.32\cdot P_{g}\cdot a_{glass}$$

(13) $$w_{cbw}\left(t\right)=0.23\cdot C_{0}\cdot a+0.32\cdot P_{g}\cdot a_{glass}$$

(14) $$Q\left(t\right)=q_{cem}\cdot C_{0}\cdot a+q_{glass}\cdot P_{g}\cdot a_{glass}$$

where $q_{cem}$ and $q_{glass}$ are the heat released by complete hydration of Portland cement and glass powder, respectively.

## 3. Results and Discussion

### 3.1. Validation

**Figure 2:**
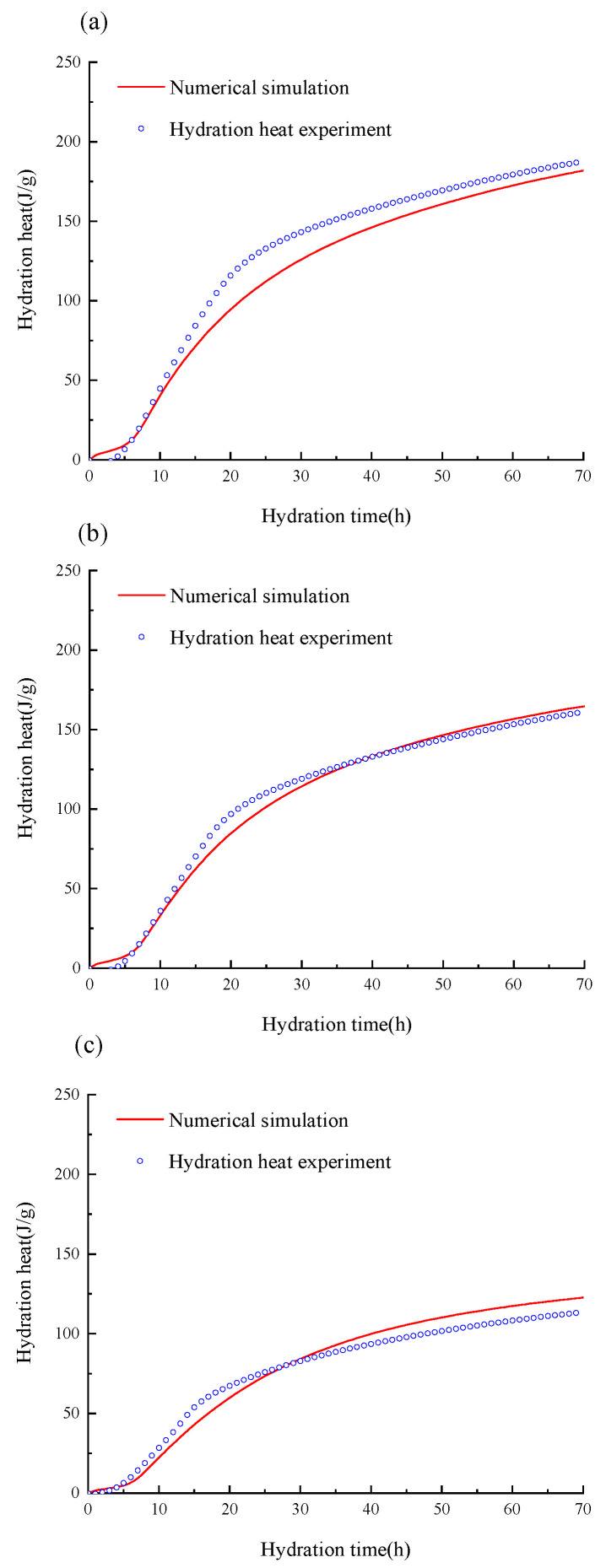
Comparison of experimental and numerical values of hydration heat under different amounts of glass powder. (**a**) ordinary cement (**b**) 20% glass powder (**c**) 50% glass powder.

**Figure 3:**
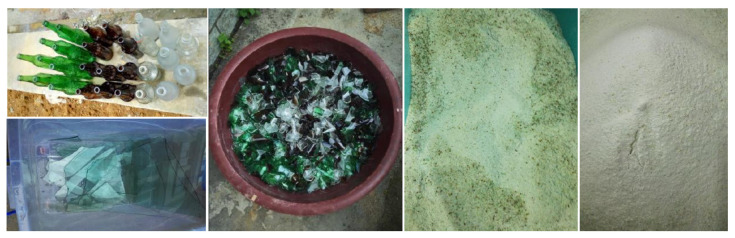
Treatment process of waste glass evolving into glass powder.

To verify the reliability of the binary hydraulic kinetics model of glass powder-cement established in this paper, the comparison between the evolution of the hydration heat with the hydration time obtained in the numerical simulation and the third-party experimental data is shown in Figure 2. It is worth noting that, in the experiment, waste glass mainly comes from waste brown beer bottles. The collected waste glass is finally formed into glass powder with the particle size of a submicron after cleaning, manual crushing, mechanical crushing, ball milling and other processes, as shown in Figure 3. Furthermore, the glass powder, cement and water are mixed, and ToniCal differential calorimeter of Germany TONI Technik Company is used to continuously measure the heat release rate of the cementitious material during hydration within 72 h, as shown in Figure 2. In the process of the experiment and numerical simulations, the water-binder ratio of the cementitious material was 0.5, the replacement rates of glass powder content are 0, 20% and 50%, respectively, and the average particle size of the glass powder was 32 μm. Table 2 shows the chemical composition of the cement and glass powder; Table 3 shows the relevant parameters used in the numerical model. It can be seen from Figure 2 that the numerical simulation is in good agreement with the experiment, which verifies the reliability of the numerical model proposed in this paper.

### 3.2. Hydration Reaction of Glass Powder-Cement Mixed Cementitious Material

**Figure 4:**
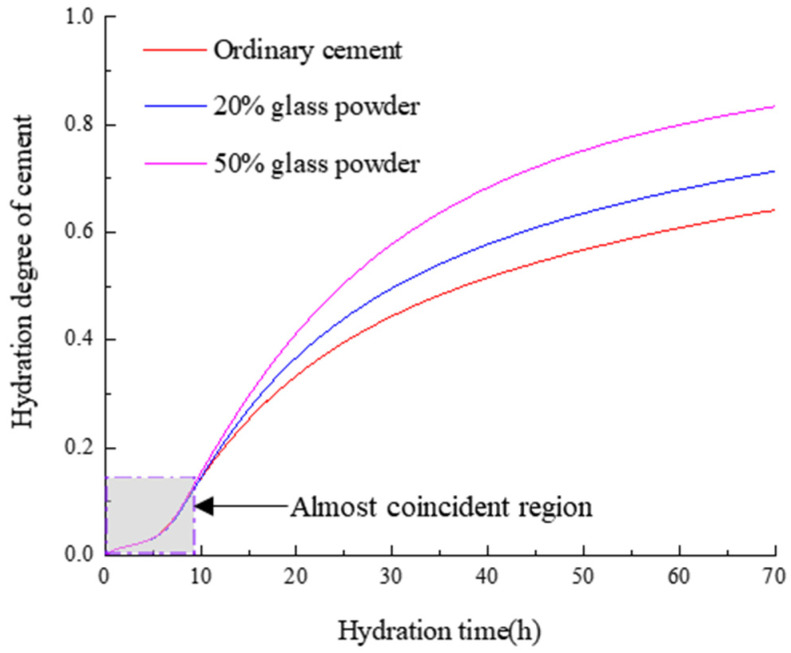
Curve of cement hydration with time.

Figure 3 shows the curve of the cement hydration degree changing with the time under three working conditions of 0, 20% and 50% glass powder content. It can be seen that with the increase in time, the higher the content of glass powder, the higher the hydration degree of the cement. Under the same water-binder ratio, the glass powder is used to replace part of the cement, the mass ratio of water to cement is increased, and the mixing of the glass powder has a dilution effect on the cement hydration. The content of capillary water in the microstructure of the cement hydration has a very important effect on the hydration of the cement. Glass powder replaces part of the cement because the hydration reaction of the glass powder is later than that of water, so at the initial stage of reaction, the free water in the capillary of the sample doped with glass powder is higher than that of ordinary cement, which promotes the hydration of the cement. At the same time, the pozzolanic reaction of the glass powder consumes part of the CH in the solution, which makes the hydration of the cement proceed in the positive direction and accelerates the hydration of the cement. In the early stage of hydration, there is a layer of water curtain on the surface of the cement particles, which hinders the diffusion of the water into the cement particles and delays the hydration of the cement. It is obvious that in the early stage of the hydration reaction, the hydration curves of the three working conditions are relatively close, as shown in Figure 4, which indicates that glass powder has little effect on the cement hydration delay.

### 3.3. Chemically Bound Water and Capillary Water Content

**Figure 5:**
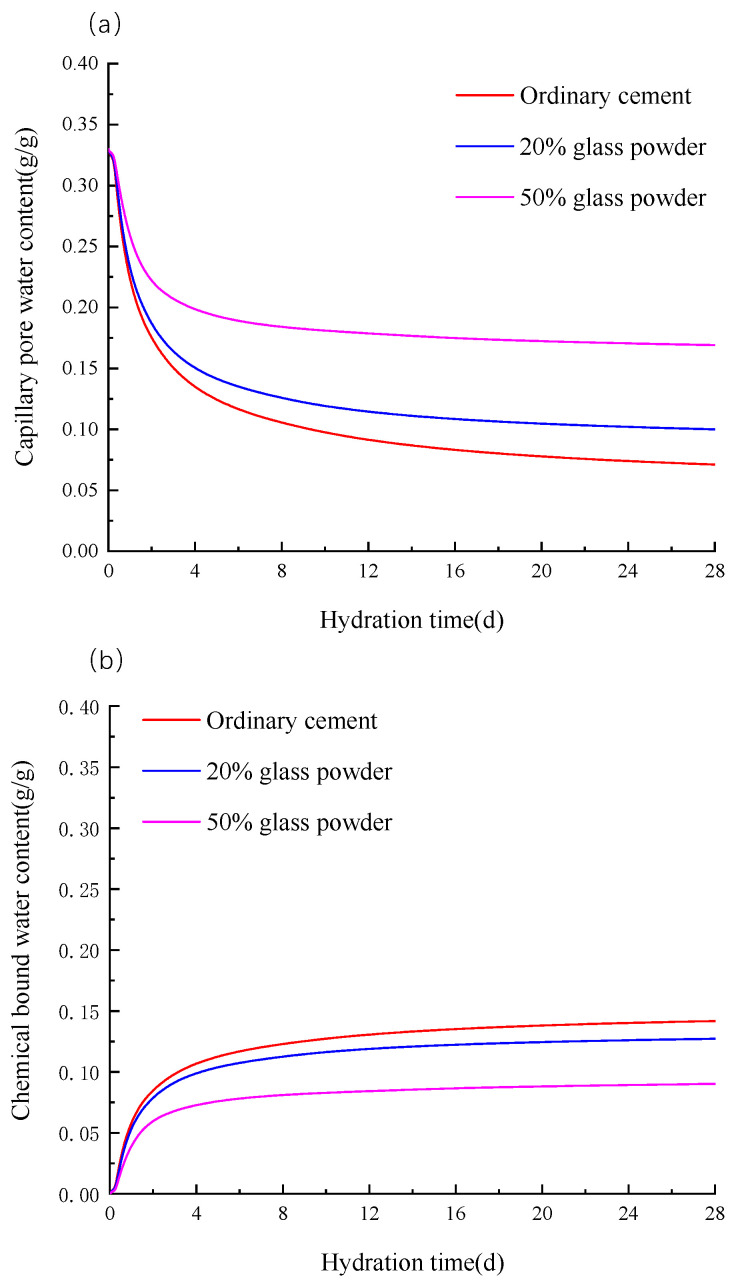
Evolution curve of capillary water and chemically combined water with hydration time. (**a**) capillary water (**b**) chemically combined water.

Chemically bound water and capillary pore water are important indicators to measure the degree of the hydration reaction, and chemically bound water is measured by the loss on ignition weight method [24]. Figure 5 shows the curves of the chemically bound water and capillary pore water changing with the time under three working conditions of glass powder dosages of 0, 20% and 50%. As the cement hydration rate is fast in the previous period, the capillary pore water of the ordinary cement decreases rapidly, and the capillary pore water is 0.07115 g/g at 28 days and tends to be stable. On the contrary, the content of chemically bound water increased rapidly, and it was 0.1475 g/g at 28 days. It can be seen from the figure that the change trend of the chemically bound water and capillary water in three working conditions with the time is the same, which increases rapidly at first, and then becomes stable in the region.

### 3.4. Analysis of Influencing Factors

The particle size and content of glass powder are the main factors affecting the hydration activity of the glass powder-cement mixed cementitious materials. Therefore, the effect of the glass particle size and substitution rate on the hydration reaction activity was studied in this paper.

#### 3.4.1. Influence of Particle Size of Glass Power

**Figure 6:**
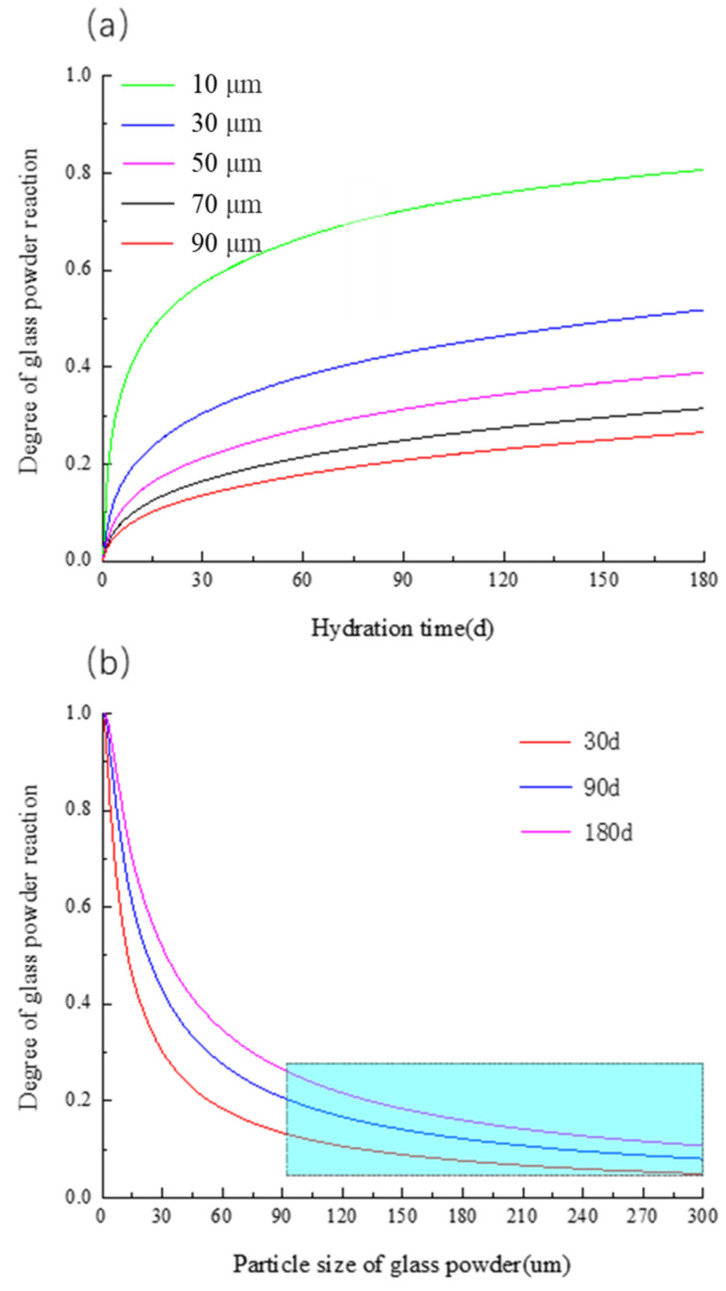
Effect of glass particle size on degree glass powder reaction (**a**) Glass powder reactivity—time curve of different glass powder sizes (**b**) Glass particle size and glass powder reactivity curve.

The smaller the glass powder particle size, the higher the pozzolanic activity [26], the larger the specific surface area, and the larger the contact area with CH, the higher the probability of reaction. The effect of the glass powder particle size on the pozzolanic reaction is analyzed by taking the glass powder content of 20% and water binder ratio of 0.5 as an example. Figure 6a is the curve of the reaction degree of glass powder with time under different particle sizes. It is evident that the finer the glass powder is, the higher the reaction degree of the glass powder is at the same curing time. Hence, the preparation of glass powder into nanoscale particles can greatly improve the activity of glass powder, but the corresponding preparation cost will also greatly increase. For example, Ali M. Onaizi et al. [27] has shown that nano glass powder has good hydration activity and can effectively improve the mechanical properties of glass concrete. Figure 6b shows the curve of reactivity of the glass powder changing with the particle size at 30 d, 90 d and 180 d, from which it can be seen that the reactivity of the glass powder decreases exponentially with the particle size. When the particle size is greater than 90 μm, the particle size has little effect on the reactivity of the glass powder. It can also be seen from the figure that when the particle size is greater than 90 μm, the reactivity of the glass powder tends to be flat at 30 d, 90 d and 180 d. When the particle size changes from 1 μm to 90 μm, the particle size change has a great influence on the particle size of the glass powder reactivity, and the reactivity of the glass powder decreases sharply with the increase in the particle size. It is noteworthy that when the glass particle size is greater than 90 μm, the reaction activity of the glass powder is less than 0.2, and the glass powder mainly acts as a filler.

#### 3.4.2. Influence of Glass Powder Content

**Figure 7:**
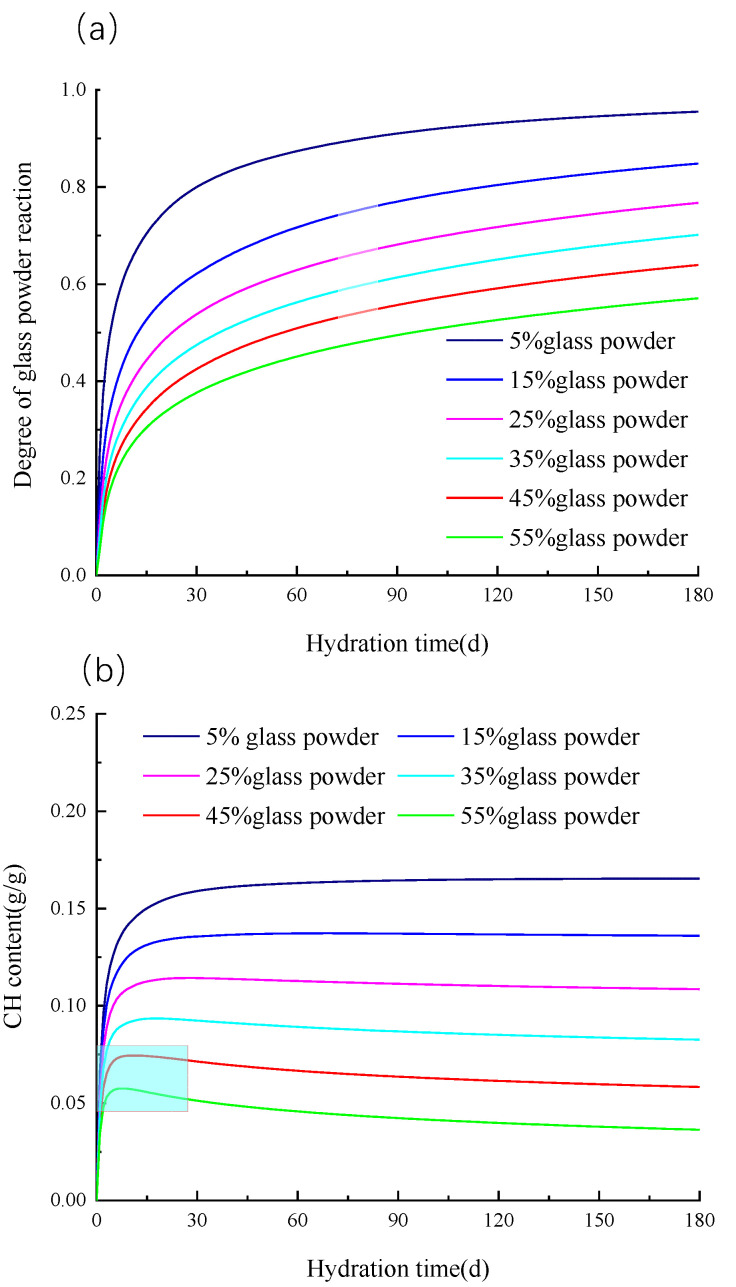
The reaction degree of glass powder and CH content changed with the reaction time. (**a**) the degree of glass powder reaction (**b**) CH content.

The glass powder content has a dilution effect on the cement hydration and accelerates the cement hydration rate. It can be seen from Figure 7a that the reactivity of the glass powder decreases with the increase in the glass powder content, which is one of the reasons for the lower early strength of the concrete with the increase in the glass powder content. As can be seen from Figure 7b, the CH content change curve shows that the CH content decreases with the increase in the glass powder content. When the content of glass powder is 45% and 55%, the CH content reaches the peak value in the early stage of hydration, which indicates that the amount of CH produced by cement hydration is less than that consumed by the pozzolanic reaction of glass powder.

## 4. Conclusions

By analogy with the slag-cement binary hydration kinetic model, the glass powder-cement binary hydration kinetic model is established, and the reliability of the proposed model is verified by third-party experiments. The following conclusions can be drawn:

1. By comparing the hydration degree of cement with ordinary cement, 20% glass powder and 50% glass powder has a dilution effect on cement hydration and accelerates cement hydration. This dilution effect is mainly caused by the hydration reaction and water diffusion; however, at the initial hydration delay stage, the dilution effect of the glass powder can be ignored.
2. The particle size of the glass powder has a great effect on the hydration of the glass powder, and the hydration degree of the glass powder decreases exponentially with the increase in the particle size. It is noteworthy that when the glass particle size is greater than 90 μm, the reaction activity of the glass powder is less than 0.2, and the glass powder mainly acts as a filler.
3. The reactivity of glass powder decreases with the increase in the glass powder content. Compared with the sample with 5% glass powder content, the hydration degree of the glass powder decreased by 42.3% for the sample with 50% glass powder content. The CH concentration in the pore solution decreases with the increase in the glass powder content, which is the reason for the decrease in the hydration degree of the glass powder with the increase in the glass powder content.
4. When the content of glass powder exceeds 45%, the CH concentration has a peak value, which indicates that the CH amount produced by cement hydration is less than the CH amount consumed by the pozzolanic reaction of the glass powder.

## Figures and Tables

**Table 1:** Consumption of various substances in pozzolanic reaction per g glass powder.

Produce Chemical. Combined Water	Consume Capillary Water	Consume CH
0.24~0.4	0.16~0.2	0.18~0.3

**Table 2:** Chemical compositions of cement and glass powder.

**Compositions**	**SiO_2_**	**Al_2_O_3_**	**CaO**	**Na_2_O**	**K_2_O**
Cement	21.56	4.78	59.64	0.21	0.85
Glass powder	70.23	3.13	8.95	13.64	0.86
**Compositions**	**Fe_2_O_3_**	**MgO**	**SO_3_**	**TiO_2_**	**Other**
Cement	3.12	2.21	3.62	0.15	3.86
Glass powder	1.37	0.78	0.05	0.10	0.89

**Table 3:** Values of numerical simulation parameters.

Parameter	Value	Parameter	Value
B_cem_	8.5 × 10^−10^	B_glass_	7.98 × 10^−8^
C_cem_	0.034	C_glass_	0.1
Kr_cem_	4.037 × 10^−6^	Kr_glass_	9.8 × 10^−7^
De_20_	4.03 × 10^−10^	De_20_	5.92 × 10^−11^

## Data Availability

The data used to support the findings of this study are included within the article.
